# S214. USING ONLINE-SCREENING TO DETECT PARTICIPANTS AT CLINICAL HIGH-RISK FOR PSYCHOSIS

**DOI:** 10.1093/schbul/sby018.1001

**Published:** 2018-04-01

**Authors:** Mhairi McDonald, Andrew Gumley, Stephen Lawrie, Matthias Schwannauer, Ruchika Gajwani, Joachim Gross, Peter Uhlhaas

**Affiliations:** 1 Institute of Neuroscience and Psychology, University of Glasgow; 2 Institute of Health and Wellbeing, University of Glasgow; 3 University of Edinburgh; 4 Institute of Neuroscience & Psychology, University of Glasgow

## Abstract

**Background:**

Identification of participants at clinical high-risk (CHR) for the development of psychosis is an important objective of current preventive efforts in mental health research. However, the utility of using web-based screening approaches to detect CHR-participants at the population-level has not been investigated.

**Methods:**

We tested a web-based screening approach to identify CHR-individuals. Potential participants were invited to a website via email-invitations, flyers and invitation letters involving both the general population and mental health services. 2121 participants completed the 16-item version of the prodromal questionnaire (PQ-16) and a 9-item questionnaire of perceptual and cognitive aberrations (PCA) for the assessment of Basic Symptoms (BS) online.

**Results:**

54% of participants met a-priori cut-off criteria for the PQ and 72 % for PCA-items online. 969 participants were invited for a clinical interview and n = 277 interviews were conducted (response rate: 29%) using the Comprehensive Assessment of At-Risk Mental State (CAARMS) and the Schizophrenia Proneness Interview, Adult Version (SPI-A). N = 88 CHR-participants and n = 8 first-episode psychosis (FEP) were detected. ROC-curve analysis revealed good to moderate sensitivity and specificity for predicting CHR-status based on online-results for both UHR- and BS-criteria (Sensitivity/Specificity: PQ-16 = 76%/50.4%; PCA = 89%/19.7%). CHR-participants were characterized by similar levels of functioning and neurocognitive deficits as clinically identified CHR-groups.

**Discussion:**

These data provide evidence for the possibility to identify CHR-participants through population-based web-screening. This could be an important strategy for early intervention and diagnosis of psychotic disorders.

